# The role of specialty palliative care interdisciplinary team members in acute care decision support: a qualitative study protocol

**DOI:** 10.1186/s12904-023-01328-9

**Published:** 2024-01-03

**Authors:** Meredith MacMartin, Jingyi Zhang, Amber Barnato

**Affiliations:** 1 Dartmouth Institute for Health Policy and Clinical Practice, Geisel School of Medicine at Dartmouth, Lebanon, NH USA; 2 Section of Palliative Care, Department of Medicine, Dartmouth Hitchcock Medical Center, Lebanon, NH USA

**Keywords:** Palliative care, Decision making, Qualitative methods

## Abstract

**Background:**

Specialty palliative care interdisciplinary teams (IDT) can play an important role in supporting patients and family members during acute care decision-making. Despite guidelines and evidence emphasizing decision-making support as a key domain of specialty palliative care, little is known about how decision-making support is actually implemented by specialty palliative care IDTs. This study aims to (1) describe the structure and processes of inpatient decision-making support delivered by specialty palliative care IDT, and (2) examine the perspectives of IDT members on their role in this decision-support.

**Methods:**

A team of clinician and non-clinician researchers will conduct non-participant observation ethnography at a single medical center in northern New England. The ethnography will focus on the work of IDT members in supporting decision making, particularly elements of specialty palliative care that have limited descriptions in the literature (e.g. systems and processes of care). Observations of formal and informal interactions between IDT members and clinical encounters will be conducted at one site over four months. Participants include patients, care partners, non-specialty palliative care providers, and specialty palliative care IDT members. Additionally, we will conduct semi-structured interviews with IDT members across three geographically diverse specialty palliative care teams across the United States to explore providers’ first-person perspective on their roles and function in decision-making support for hospitalized patients. Field notes and transcripts from observation and interviews will be uploaded to Dedoose software for management and thematic analysis following an inductive approach.

**Discussion:**

To our knowledge, this will be the first observational study of the roles of interdisciplinary specialty palliative care teams. Results from this research will support further investigation into implementation of decision-making support across different types of medical teams.

## Background

Although decision support is a distinguishing feature of specialty palliative care, [1–4] we know little about the structures or processes that support it among inpatient interdisciplinary teams (IDT). The centrality of decision-making support to palliative care practice is evident in the literature and throughout the Clinical Practice Guidelines from the National Consensus Project (NCP) [1–5]. To date, studies of this type of decision support delivered by specialty palliative care teams have focused on identifying patient preferences, characterizing patient participation, and quantifying the outcomes of decision making. The unit of analysis has most often been the clinician-patient dyad, with little to no investigation into the contributions of the interdisciplinary team to the process [6].

Like decision-making support, the centrality of an IDT is emphasized throughout the NCP practice guidelines, which make it clear that palliative care is “provided by a team of physicians, advanced practice registered nurses, physician assistants, nurses, social workers, chaplains, and others based on need” (Guideline 1.1) [5]. The importance of the IDT is also demonstrated in many of the clinical trials of palliative care interventions, in which patients received care not just from palliative care physicians, but nurses, social workers, and chaplains. Detailed studies of IDT collaboration have been conducted in hospice teams, [7, 8] but little investigation has been done into the processes and behaviors of non-hospice palliative care teams. There has been some study of multidisciplinary and interdisciplinary support of advance care planning (ACP) and shared decision making (SDM) particularly in the intensive care unit; [9–11] however, the question of how specialty palliative care interdisciplinary teams support decision making in the hospital remains unanswered.

As efforts are ongoing to operationalize the critical components of specialty palliative care, understanding how IDTs implement the key domain of decision-making support is a critical need. To fill this gap, we aim to use ethnographic methods to generate a description and classification of how specialty palliative care teams support decision making in the hospital. To our knowledge, this will be the first such investigation into the role of the full interdisciplinary team in this core component of specialty palliative care.

## Methods/design

### Overall study design

We chose ethnographic methods for this study as these research methods are best suited for generating descriptions of complex phenomena (Table 1) [12]. In particular, ethnographic methods are well suited to examine structures and processes of decision support in clinical practice that may be difficult to examine with other methods such as chart review. Interviews are well suited to examine first-person perspectives and may uncover subtle or unspoken beliefs or perceptions regarding the role of IDT members in supporting decision making.

**Table 1:** Data collection rationale

Data Collection Method	Rationale
Ethnography	To examine processes and behaviors of decision support in clinical practice that may be difficult to examine with other methods such as chart review.
Semi-structured provider interviews	To examine first-person perspectives and uncover subtle or unspoken norms and practices regarding the role of IDT members in supporting decision making

This study will be conducted in two phases. First, we will conduct non-participant observation of decision-making support provided by a single interdisciplinary inpatient specialty palliative care team at a single academic medical center in northern New England, to identify the ways in which the full team, particularly the non-medical providers, support decision making. Second, we will conduct semi-structured interviews with members of interdisciplinary specialty palliative care teams at three geographically diverse institutions to assess the roles of palliative care team members in decision-making support and institutional and discipline-specific practices around decision support.

We will report our findings according to the Consolidated Criteria for Reporting Qualitative Research (COREQ) [13].

### Conceptual framework

We define decision-making support as the process(es) by which palliative care specialists and teams assist patients and families in making a healthcare decision at the time the decision presents itself, which has been labeled “in the moment” decision making by scholars in the field. “In the moment” decision making is distinguished from decision making done in advance via ACP. For example, a patient hospitalized with complications from cancer progression despite active treatment is faced with an "in the moment" decision between initiating next-line chemotherapy or discontinuing cancer-directed therapy to enroll in hospice.

We will use the paradigm of SDM as a conceptual foundation for this proposed work seeking to understand processes of interdisciplinary decision-making support. We will use the “Three Talk” model of SDM as a starting place, in which “Team Talk” consists of indicating choice, providing support, and identifying goals; “Option Talk” consists of comparing alternatives and discussing harms and benefits; and “Decision Talk” consists of getting to informed preferences and making preference-based decisions [14].

### Phase I: ethnography

#### Study population and recruitment

There are three primary populations that we will include in this phase. They are (1) members of the interdisciplinary specialty palliative care team; (2) non-palliative care clinicians caring for palliative care patients (primary teams); and (3) palliative care patients and their care partners (Table 2).

**Table 2:** Ethnography study populations, eligibility, recruitment strategies, and consent procedures

Study population	Eligibility	Recruitment strategy	Consent procedure
Palliative care clinicians	All members of the palliative care clinical team, except for volunteers	Approach during scheduled section meeting and via email	Written consent
Non-palliative care clinicians	All members of clinical teams caring for patient participants	Contact likely participants ahead of time by email	Verbal consent, written information to be provided
Patients and family	Those for whom the palliative care team is providing decision-making support, identified by (1) having a discrete decision to make and (2) discussing that decision with members of the palliative care team	Screened during daily palliative care IDT meetings and in discussion with members of the palliative care team	Verbal consent, written information to be provided
Medical staff, patients, and families not directly participating	N/A	N/A	Written information to be provided if possible

#### Qualitative data collection

The study team will collect four types of data: (1) transcripts of interdisciplinary team meetings relating to decision support, (2) transcripts from non-patient facing clinical encounters such as informal meeting planning with a referring team in advance of a patient visit, (3) transcripts from patient-facing clinical encounters such as family meetings, and (4) field notes from direct observation of each type of encounter. We will link data from related observations (meeting planning and then family meeting, for example) using a unique identification number (ID). We will record and transcribe interdisciplinary team meetings using the native recording and transcription function in the videoconferencing software used by the palliative care team for IDT meetings. We will record, when feasible, non-patient facing and patient-facing clinical encounters using a handheld audio recorder. Recordings will be transcribed securely using a professional transcription service.

We will collect field notes in a semi-structured fashion, and each observation will be linked with a unique ID to the participants in the encounter. In addition to encounter participants, we will include documentation of the type of encounter. We will note aspects of encounters that cannot be adequately captured by audio-recording, such as body posture, eye contact, and positioning within the room. We will collect clinician demographics via a clinician-completed survey. We will collect patient demographics in person at the time of clinical encounters.

#### Qualitative data analysis

We will use a practical thematic approach to analysis of the raw data [15]. We will use Dedoose, a qualitative data analysis platform, to manage and analyze all transcriptions and digitized field notes. We will carry out an immersive review of the raw data and analytic memoing, followed by coding of a subset of data to develop an initial coding guide. We will then use the initial coding guide to analyze subsequent data, with iterative development of codes and then identification and refinement of themes. Following elaboration of themes, we will map our findings onto the “Three Talk” model of SDM and revise that model as appropriate.

### Phase II: interviews

#### Study population and recruitment

We have used professional networks to identify two collaborating sites in the United States. Each site has a well-established specialty palliative care team and a National Cancer Institute (NCI) Comprehensive Cancer Center. Collaborating sites were selected to achieve geographic diversity (Southeast and Western United States in combination with our home institution in the Northeastern US) and to ensure diversity in the patient populations represented as our collaborating sites serve significant populations of racially minoritized patients. We will recruit members of the interdisciplinary specialty palliative care team from all three partnering institutions. We will use a stratified sampling strategy to recruit participants to ensure all the major disciplines within a palliative care team are represented, including physicians, advanced practice providers (APPs), nurses, social workers, and chaplains/spiritual care providers. We will sample from each category at each site to achieve an estimated total sample size for each site of 8–10 participants with at least one respondent from each category (Table 3). The estimated total sample size of 25–30 is based on average sample size reported in previous qualitative literature and is aimed to achieve thematic saturation [16, 17].

**Table 3:** Target sampling frame for interviews

Data collection setting	Sample	N
Ethnography	Patients	40
	Care partners	40
	Non-palliative care providers	50–60
	Palliative care providers	34
Interviews	Palliative care providers (from each site)	8–10
	Physicians	At least 1
	Advanced practice providers (APPs)	At least 1
	Nurses	At least 1
	Social workers	At least 1
	Chaplains/Spiritual care providers	At least 1

#### Qualitative data collection

We will develop a semi-structured interview guide incorporating findings from the work described in the ethnography phase. Anticipated topics in the interview guide include: (1) perceived role and/or responsibility for supporting decision making within the IDT; (2) barriers and facilitators to carrying out that role; and (3) satisfaction with role in supporting decision making. We will pilot test the interview guide with a convenience sample of palliative care interdisciplinary team members from non-study sites. Participants will complete a short questionnaire assessing demographic characteristics, which will not be used to evaluate differences between sites or clinical roles, but rather to set the context. We will conduct all interviews in person or by videoconferencing such as Zoom, record the interviews, transcribe them professionally, and de-identify them before uploading them to Dedoose, a secure, web-based platform for data management and qualitative analysis, for analyzing.

#### Qualitative data analysis

As with Phase I, we will use a practical thematic analysis approach. We will carry out an immersive review of the transcripts with analytic memoing, followed by open coding of a subset of data to develop an initial coding guide. We will then use the initial coding guide to analyze subsequent data, with iterative development of codes and then identification and refinement of themes.

### Rigor and reproducibility

Our research team will use multiple methods to establish trustworthiness through the process of collecting and analyzing data. Our research team (N = 3) is composed of one palliative care physician, one PhD-prepared research scientist, and a research assistant. To maximize credibility, dependability, transferability, and confirmability, each observer will keep a journal dedicated to documenting logistics of observation, decisions about the research and rationales, and reflections on internal dialogue and personal perspectives about the data being gathered. We will conduct peer debriefing within the research team and engage in researcher triangulation amongst ourselves and with qualitative methods mentors. We will maintain thorough and detailed documentation throughout the research process including an audit trail of code and theme development and methodological and analytical choices. Finally, we will conduct member checking with participants from the interdisciplinary palliative care team for the themes developed in analysis.

## Discussion

This is the first qualitative ethnography and interview study about the role of the interdisciplinary team in decision-making support both within and outside of the provider-patient encounter. If our study aims are achieved, we expect to generate a set of themes that describe the ways in which a specialty interdisciplinary palliative care team supports medical decision making in the hospital and a thick description of the perspectives of members of IDTs at multiple institutions and in multiple clinical roles. We also anticipate adapting the “Three Talk” conceptual model to reflect interdisciplinary decision support processes.

Despite the prominent role of decision-making support in palliative care clinical practice and amid calls to shift from decision-making support in advance (advance care planning) to at the time of the decision (“in the moment”), [18] guidelines for *how* to implement this kind of decision-making support have not been developed. Insights derived from this work will provide the foundation for further investigation into the implementation of decision support practices across institutions and how such support correlates with patient outcomes. This in turn will support two longer-term goals. The first is to improve the implementation of interdisciplinary decision-making support provided by specialty palliative care teams, both in terms of the quality of the support provided and the reach of support to patient populations in need. The second goal is to develop models of interdisciplinary decision-making support that can be provided by teams outside of specialty palliative care. The need to support patients making “in the moment” decisions is certainly not confined to specialty palliative care; indeed these are situations that affect nearly every clinician across all specialties. We hope to leverage the expertise and experience of specialty palliative care to gain insight into the ways teams of healthcare providers help patients and families in these difficult circumstances, with the goal of identifying best practices in supporting “in the moment” decision making that could be exported to other clinical specialties.

## Data Availability

After completion of this study, the dataset collected will be available through the Palliative Care Research Cooperative QDR-EOLPC (https://data.qdr.syr.edu/dataverse/pcrc).
